# Use of whole genome sequences to develop a molecular phylogenetic framework for *Rhodococcus fascians* and the *Rhodococcus* genus

**DOI:** 10.3389/fpls.2014.00406

**Published:** 2014-08-19

**Authors:** Allison L. Creason, Edward W. Davis, Melodie L. Putnam, Olivier M. Vandeputte, Jeff H. Chang

**Affiliations:** ^1^Department of Botany and Plant Pathology, Oregon State UniversityCorvallis, OR, USA; ^2^Molecular and Cellular Biology Program, Oregon State UniversityCorvallis, OR, USA; ^3^Laboratoire de Biotechnologie Vegetale, Universite Libre de BruxellesGosselies, Belgium; ^4^Center for Genome Research and Biocomputing, Oregon State UniversityCorvallis, OR, USA

**Keywords:** Gram-positive, plant pathogen, prokaryotic taxonomy, leafy gall, average nucleotide identity

## Abstract

The accurate diagnosis of diseases caused by pathogenic bacteria requires a stable species classification. *Rhodococcus fascians* is the only documented member of its ill-defined genus that is capable of causing disease on a wide range of agriculturally important plants. Comparisons of genome sequences generated from isolates of *Rhodococcus* associated with diseased plants revealed a level of genetic diversity consistent with them representing multiple species. To test this, we generated a tree based on more than 1700 homologous sequences from plant-associated isolates of *Rhodococcus*, and obtained support from additional approaches that measure and cluster based on genome similarities. Results were consistent in supporting the definition of new *Rhodococcus* species within clades containing phytopathogenic members. We also used the genome sequences, along with other rhodococcal genome sequences to construct a molecular phylogenetic tree as a framework for resolving the *Rhodococcus* genus. Results indicated that *Rhodococcus* has the potential for having 20 species and also confirmed a need to revisit the taxonomic groupings within *Rhodococcus*.

## Introduction

Defining bacteria into stable and coherent genetically similar species has many practical implications. However, multiple factors including effective population size, horizontal gene transfer and bacterial recombination, and their barriers, affect cohesiveness of different groups of bacteria to varying degrees (Doolittle and Zhaxybayeva, 2009). As a consequence, a unifying concept for bacterial species has yet to be adopted, which has made it difficult to develop criteria and thresholds that can be generally applied for defining bacterial species.

Traditional polyphasic approaches define bacterial species as a monophyletic group with at least one discriminative phenotypic trait. Though pragmatic and widely adopted, the traditional approaches are weighted toward phenotypic traits and cannot keep pace with the rate in which new genotypes are being discovered and sequenced. With major advances in contemporary methods in sequencing, operational criteria based on whole genome sequences have been developed and adopted to assist in resolving bacterial phylogeny (Konstantinidis et al., 2006). Multi-locus sequence analysis (MLSA) and trees based on whole genome sequences are powerful methods for inferring evolutionary relationships (Staley, 2009). Alternative criteria based on the degree of similarities in genome signatures have also been developed (Konstantinidis et al., 2006; Bohlin et al., 2008; Richter and Rosselló-Móra, 2009). Average nucleotide identity (ANI), for example, is a simple measure of genetic relatedness based on sequences conserved among compared genomes and has gained acceptance as a method for defining bacterial species (Konstantinidis et al., 2006; Chan et al., 2012; Kim et al., 2014). ANI has also been developed as a method for codifying bacteria based on genome similarity (Marakeby et al., 2014).

Genome-enabled comparisons and the recognition of environmental niches in structuring gene flow have revealed a diversity of population structures for groups of plant-associated bacteria. *Pseudomonas fluorescens*, for example, occupies multiple niches and has a level of heterogeneity consistent with limited gene flow between sub-clades that challenge their taxonomy (Loper et al., 2012). Likewise, a change to the taxonomy of *Agrobacterium tumefaciens* has been proposed to reflect genome-enabled discovery of clade-specific traits (Lassalle et al., 2011). The ANI method has been used to assign newly discovered isolates to known plant-associated species and discover new species of plant pathogens (Dudnik et al., 2014; Durán et al., 2014; van der Wolf et al., 2014).

The Gram-positive *Rhodococcus* genus is a member of the *Nocardiaceae* family and forms a distinct group with 30 valid species published (Jones and Goodfellow, 2012). The genus has diverse members that inhabit a wide range of terrestrial as well as aquatic habitats and are renowned for their catabolic functions and ability to degrade a large number of organic compounds (Larkin et al., 2005). Additionally, members of *Rhodococcus* have been recovered from extreme environments such as the deep-sea, oil-contaminated soils, and freeze-thaw tundra on glacial margins (Sheng et al., 2011; Shevtsov et al., 2013; Konishi et al., 2014). Because of their biotechnological applications and potential in bioremediation, there has been a dramatic increase in the number of sequenced *Rhodococcus* genomes. Their genomes are high in GC content and range in size from 4.3 megabases (Mb) to over 10 Mb. Most genomes are larger than 5 Mb and their large sizes have been attributed to both horizontal gene transfer and gene duplication (Letek et al., 2010). Partly due to the historical reliance on phenotypic traits and use of 16S rDNA sequence information, the *Rhodococcus* phylogeny still remains poorly resolved (Gürtler et al., 2004).

To date, *Rhodococcus fascians* and *Rhodococcus equi* are the only two members of the genus that are well documented as being pathogenic (von Bargen and Haas, 2009; Stes et al., 2011). *R. fascians* can infect a broad range of plants. After breaching the plant cuticle, the pathogen collapses the epidermal layer, and forms ingression sites beneath epiphytic colonies (Cornelis et al., 2001). *R. fascians* then grows inside the host tissue and provokes cell differentiation and *de novo* organogenesis, resulting in proliferations and abnormal growths called witches' brooms or leafy galls (Putnam and Miller, 2007). To gain insights into the mechanisms and evolution of virulence, we determined the genome sequences for 20 isolates of *Rhodococcus* (Creason et al., 2014). Like *R. equi*, *R. fascians* has few horizontally-acquired virulence genes, which are predicted to be augmented by co-option of core genes, that contribute to the ability of the bacterium to infect and cause disease (Crespi et al., 1992; Letek et al., 2008, 2010; Creason et al., 2014). Because of this mechanism of virulence evolution, phytopathogenicity is not expected to be a distinguishing trait suitable for classifying these *Rhodococcus* isolates.

In this study, we tested the hypothesis that leafy gall disease is caused by members of multiple species of *Rhodococcus*. Results from four independent methods were consistent and supported the hypothesis. Analysis of the 20 genome sequences showed the isolates formed two well supported clades, with one consisting of 16 isolates and having complex substructure indicative of multiple species. Analysis of the *Rhodococcus* genus associated four isolates collected from extreme environments or found in association with healthy plants to the two clades of *Rhodococcus* with plant-pathogenic members. Lastly, the need for revision of taxonomic grouping in *Rhodococcus* is suggested, as determined based on ANI distances calculated for all possible pairwise comparisons between members with available genome sequences.

## Materials and methods

### Isolation of phytopathogenic *Rhodococcus*

Symptomatic tissue of *Leucanthemum* × superbum “Becky,” received by the Oregon State University Plant Clinic, was washed, macerated in sterile saline, and incubated at room temperature for 30 min. *Rhodococcus* cells were selected for by culturing on semi-selective D2 media (Kado and Heskett, 1970). Isolate A22b was selected and verified as phytopathogenic based on its ability to cause leafy gall disease on pea seedlings and positive amplification for the *fasA* gene.

### Nucleic acid preparations

A22b was grown in LB at 28°C with shaking (Bertani, 1951). Genomic DNA from A22b was extracted from cells grown directly from stocks. The Wizard Genomic DNA Purification Kit was used, according to the instructions of the manufacturer, to extract genomic DNA (Promega Corporation, Madison, WI, USA).

### Next-generation sequencing, assembly, and annotation

Library construction and sequencing on an Illumina MiSeq were done in the Center for Genome Research and Biocomputing at Oregon State University. The A22b genome was assembled using Velvet (v1.2.08), with a hash length of 125 (Zerbino and Birney, 2008). The insert size was determined based on the estimated fragment size of the library preparation. Multiple assemblies were done, in which coverage cutoff, expected coverage, and hash length parameters were changed (Creason et al., 2014). The highest quality assembly was identified based on the number of contigs and having a sum total size between 5 and 6 Mb. Contigs were reordered using the genome sequence of *R. fascians* A44a as a reference and the Mauve Contig Mover (Rissman et al., 2009). The genome was annotated using Prokka (Seemann, 2014). As part of the Prokka pipeline, CDSs were annotated in part, based on BLASTP analysis and a database of genomes core to the *Rhodococcus* genus, including whole-genome assemblies from *Rhodococcus jostii* RHA1, *Rhodococcus opacus* B4, *Rhodococcus erythropolis* PR4, *R. equi* 103S, and the *R. fascians* linear plasmid, pFiD188 (Na et al., 2005; McLeod et al., 2006; Sekine et al., 2006; Letek et al., 2010; Francis et al., 2012). The whole genome shotgun project for A22b has been deposited at DDJB/EMBL/GenBank under the accession JOKB00000000 (BioProject PRJNA252927, BioSample SAMN02864791). The version described in this paper is version JOKB01000000. The A22b short reads and annotated genome sequences are available for download (SRS641819, http://dx.doi.org/10.7267/N9PN93H8). In order to be consistent, publicly available wgs sequences used in this study were similarly annotated. Their genome annotations are available upon request.

### Phylogenetic analyses

We used Hal (−a muscle and −y 100 settings) to construct the multi-gene tree of the 20 isolates and the *Nocardia farcinica* type strain as the outgroup (Ishikawa et al., 2004; Robbertse et al., 2011).

Sequences for the maximum-likelihood MLSA tree were gathered from the NCBI nt and wgs databases, using FtsY, InfB, RpoB, RsmA, SecY, TsaD, and YchF from *Rhodococcus jostii* RHA1 and *Bifidobacterium longum* subsp. *infantis* ATCC 15697 as queries in TBLASTN+ (v2.2.29) searches (with default settings; Adékambi et al., 2011). The query sequences were selected to provide coverage of the Actinobacteria phylum. Duplicate results from the two TBLASTN+ (v2.2.29) results, and strains in which all seven translated sequences were not detected, were filtered out. A total of 1316 strains passed filter.

The filtered sequences were aligned using the L-INS-i algorithm in MAFFT (v7.149b) with the –legacygappenalty flag. Gblocks (v0.91b) was used to trim the alignments prior to concatenation with half gapped positions allowed (−b5 = h setting; Castresana, 2000). Concatenated sequences with 100% identity, excluding those in the *Rhodococcus* genus, were collapsed into one entry, resulting in 961 sequences as input for tree generation. The most appropriate models of substitution for each gene were selected using the ProteinModelSelection.pl script provided with RAxML (v8; Katoh and Standley, 2013; Stamatakis, 2014). Trees were generated using RAxML (v8), based on the guidelines provided in the users manual (Stamatakis, 2014). Briefly, five starting parsimony trees were generated using the −y option; fixed initial arrangements were run on the five trees separately with the −i 10 setting. Automatic initial arrangements were also run on the five trees. The best log likelihood scores were used to choose the proper initial arrangement setting for further tree generation (−i 10 was the best for the dataset). A total of 500 rapid bootstraps (−x setting with −N 500) were performed on this dataset, and 10 distinct (−f d setting with −N 10) trees were generated. Bootstrap values were mapped on the best of the 10 distinct trees using the −f b setting. See Supplemental Data 1 for the full tree, accession values, and duplicated sequences that were removed.

Alignments were visualized using Belvu (Sonnhammer and Hollich, 2005). Images were generated using the iTOL (Letunic and Bork, 2011).

### Bioinformatic analyses

The progressiveMauve (v2.3.1) alignment was produced using default settings and as input, the chromosomal sequences for isolates D188, A21d2, 05-339-1, and A44a (Darling et al., 2010).

JSpecies with whole genome FASTA sequences as input, was used to calculate average nucleotide identities (BLAST; ANIb) and do pairwise comparisons of tetranucleotide frequencies (TETRA; Richter and Rosselló-Móra, 2009). Codon usage tables were constructed using EMBOSS cusp and sum difference statistics were calculated using EMBOSS codcmp (default settings; http://emboss.sourceforge.net/apps/cvs/emboss/apps/cusp.html). Reciprocal best BLASTP analysis was done according to methods previously reported (Creason et al., 2014).

The ANI values used to generate the distance dendrogram were calculated using published methods (Konstantinidis and Tiedje, 2005). The following were automated using *ad-hoc* scripts. Genome sequences were split into 1020-nucleotide long segments. The genome segments were used as queries in BLASTN+ (v2.2.27) searches against all other complete genomes in an all-by-all pairwise analysis. BLASTN+ (v2.2.27) was used, with the extra settings, “blastn –task blastn –dust no –xdrop_gap 150 –penalty −1 –reward 1 –gapopen 5 –gapextend 2,” for the searches (Camacho et al., 2009). Sequences with less than 70% coverage and 30% identity were filtered out, the number of results above the cutoffs were counted, and the average nucleotide identity of the resulting sequences were calculated. Results were comparable to those calculated using jSpecies and were better at handling the larger number of samples (Richter and Rosselló-Móra, 2009).

The distance dendrogram was generated using the all-by-all pairwise ANI divergence values as input, which is defined as 100%—ANI (Chan et al., 2012). The hcluster Python package was installed along with all dependencies (http://scipy-cluster.googlecode.com/). IPython, in interactive mode, was used to generate the dendogram (Perez and Granger, 2007). The matplotlib library was also required (ipython –matplotlib; Hunter, 2007; http://matplotlib.org). The pdist() function from hcluster was used to calculate the Euclidean distance between the ANI divergence values, and the complete linkage on the distance matrix was calculated using the complete() function. The dendrogram was generated using the dendrogram() function of hcluster. Input data and the resulting script from the interactive IPython session can be found in Supplemental Data 2.

Graphs were generated in R (R Core Team, 2013). The 3-D scatter plot was generated using plot3d{rgl} and quads3d{rgl}. Heatmaps were generated using heatmap.2{gplots}.

## Results

### Whole genome-based phylogeny supports multiple lineages of plant pathogenic *Rhodococcus*

In our first sequencing effort, we used hybrid approaches to generate high quality assemblies for isolates D188 and A44a (Creason et al., 2014). Unexpectedly, initial attempts to align the genome sequences were challenging, leading us to hypothesize that the two isolates represented different species of *Rhodococcus*. In order to test this hypothesis, we determined the genome sequences for 18 additional isolates of *Rhodococcus* identified from diseased plants or initially typed as *R. fascians* (Table 1; Miteva et al., 2004). The alignment of four genome sequences shows conservation of collinear blocks, with A44a being the most disparate in respect to the level of conservation and number and size of gaps between blocks (Figure S1).

**Table 1:** **Isolates of *Rhodococcus* selected for whole genome sequencing**.

Isolate^*^	**Source**	**Geographic location**	**Year^§^**	**Group^†^**
GIC26	Greenland glacial ice core	Greenland	>120,000 years	Sub-clade i
GIC36	Greenland glacial ice core	Greenland	>120,000 years	Sub-clade i
05-561-1	*Lavandula angustifolia* “Violet Intrigue”	Washington, USA	2005	Sub-clade i
LMG3605	*Chrysanthemum* × *morifolium*	United Kingdom	Unknown	Sub-clade i
*D188*	*Chrysanthemum* × *morifolium*	Europe	1984	Sub-clade i
LMG3602	*Lilium longiflorum*	Moerbeke, Belgium	Unknown	Sub-clade i
**LMG3623 (Tilford's strain)**	*Lathyrus odoratus*	USA	Unknown	Sub-clade i
A3b	*Heliopsis helianthoides* “Loraine Sunshine”	Michigan, USA	2005	Sub-clade i
LMG3616	*Lathyrus odoratus*	United Kingdom	Unknown	Sub-clade i
A78	*Leucanthemum* × *superbum* “Becky”	Pennsylvania, USA	2002	Sub-clade i
A21d2	*Oenothera speciosa* “Siskiyou”	Michigan, USA	2002	Sub-clade ii
04-516	*Aster* × “Woods Pink”	Florida, USA	2004	Sub-clade ii
A25f	*Nemesia* × “Natalie”	Washington, USA	2002	Sub-clade ii
LMG3625	*Lathyrus odoratus*	United Kingdom	1958	Sub-clade ii
05-339-1	*Hosta* “Blue Umbrellas”	Michigan, USA	2005	Sub-clade iii
A76	*Veronica spicata* “Royal Candles”	Michigan, USA	2002	Sub-clade iii
*A44a*	*Veronica spicata* “Minuet”	Oregon, USA	2002	Clade II
02-815	*Campanula* × “Sarastro”	Michigan, USA	2002	Clade II
02-816c	*Viola* × “Purple Showers”	Michigan, USA	2002	Clade II
A73a	*Aster amellus* “Violet Queen”	Pennsylvania, USA	2003	Clade II
A22b	*Leucanthemum* × superbum “Becky”	Washington, USA	2002	Clade II

* Isolates designated with LMG were obtained from Belgium co-ordinated collection of micro-organisms (BCCM); GIC isolates are from a Greenland glacial ice core; remaining isolates were obtained from diseased plants submitted to the Oregon State University (OSU) Plant Clinic. Italicized isolates, first sequenced using a hybrid approach; bold, type strain.

§ Year deposited (BCCM), isolated (OSU plant clinic), or trapped in ice (GIC isolates).

† Group designation is based on this study; sub-clades i-iii all belong to Clade I.

**Figure 1:**
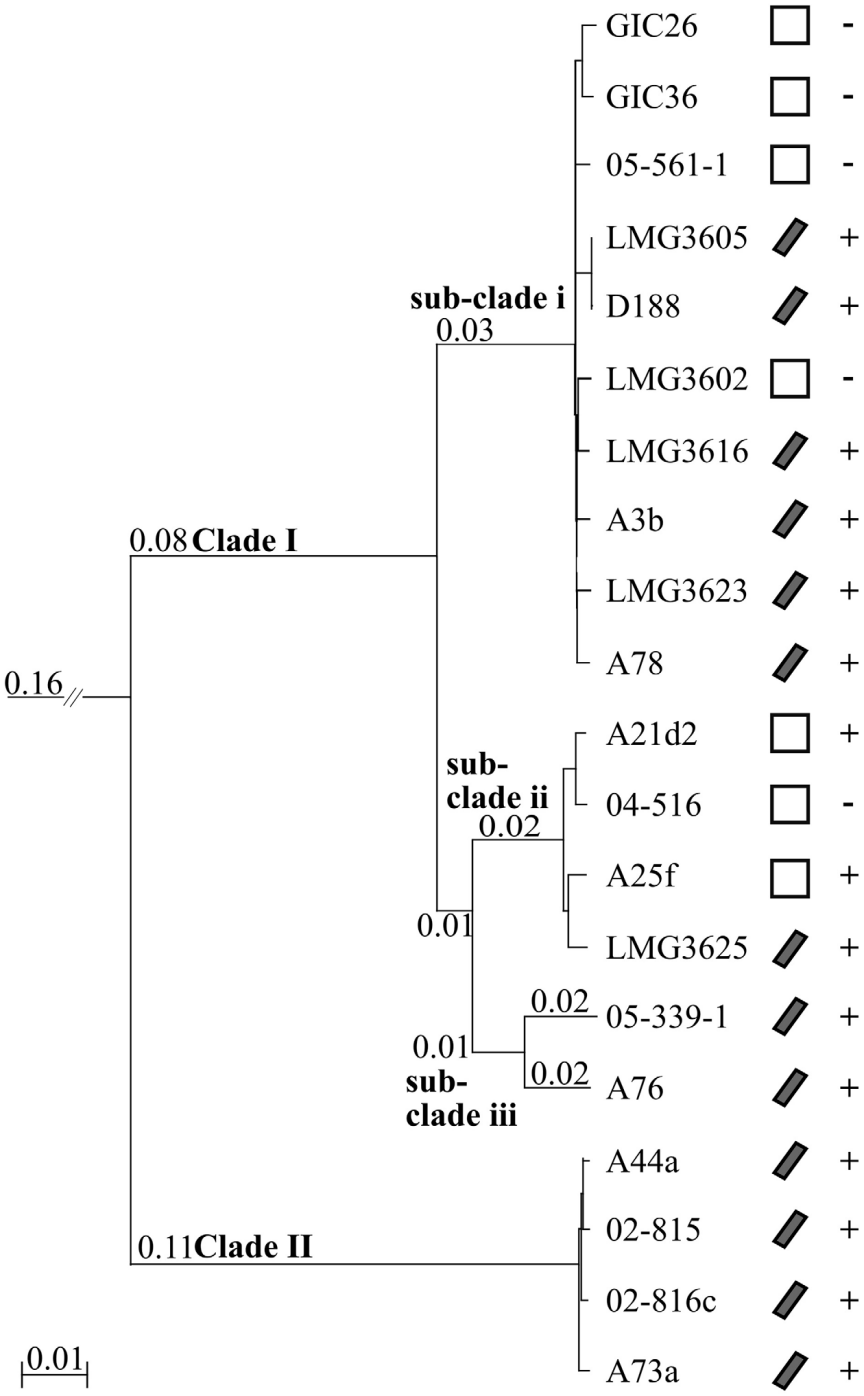
**Neighbor-joining tree based on 1727 homologous genes.** A rooted neighbor-joining tree was constructed using translated sequences from 1727 genes present in all 20 isolates and the *Nocardia farcinica* type strain (not shown). Clade and sub-clade designations are indicated at the corresponding node. Scale bar, number of amino acid substitutions per site; only branches with lengths greater than zero are indicated. Isolates with a linear plasmid are denoted with a diagonal bar; empty boxes, absence of linear plasmid. Phytopathogenic isolates are indicated with, “+”; non-pathogenic isolates are indicated with, “−.”

We constructed a multi-gene phylogenetic tree for the 20 isolates of *Rhodococcus* based on the whole genome sequences to infer the evolutionary relationships. Homologous sequences were identified from all 20 and from the type strain of *Nocardia*, which we used as an outgroup (Ishikawa et al., 2004). Clusters with paralogs, but not those with sequences potentially acquired via recombination, were filtered out, leaving 1727 clusters. The corresponding sequences from each isolate were concatenated, aligned, and used to derive a neighbor-joining (NJ) tree. The isolates formed two distinct clades (Figure 1). Clade I has substructure, with the largest and deepest branching sub-clade i consisting of the type strain LMG3623, D188, the two glacial ice core isolates, three other culture collection isolates, and three isolated from diseased plants. Sub-clade ii includes three isolates obtained directly from diseased plants and one obtained from a culture collection. Sub-clade iii is represented by just two isolates, 05-339-1, and A76, collected from diseased plants. As indicated by the longer branch lengths, the two isolates are more diverged. Clade II consists of four isolates from the US that includes A44a.

We projected the presence/absence of a linear virulence plasmid and trait of phytopathogenicity on to the phylogenetic tree (Figure 1; Creason et al., 2014). Phytopathogenicity is not exclusive to any clade or sub-clade. The five non-pathogenic isolates clustered in Clade I with four and one found in sub-clades i and ii, respectively. Sub-clade ii is the most variable in respect to virulence loci structure, as phytopathogenic A21d2 and A25f lack a linear virulence plasmid and A21d2 also lacks the entire *fas* operon (Creason et al., 2014). These data are consistent with our hypothesis that leafy gall disease is caused by multiple species of *Rhodococcus* and explain why initial attempts in aligning the whole genome sequences of D188 and A44a were difficult.

### Alternative whole-genome based analyses support the multiple lineages of *Rhodococcus*

**Figure 2:**
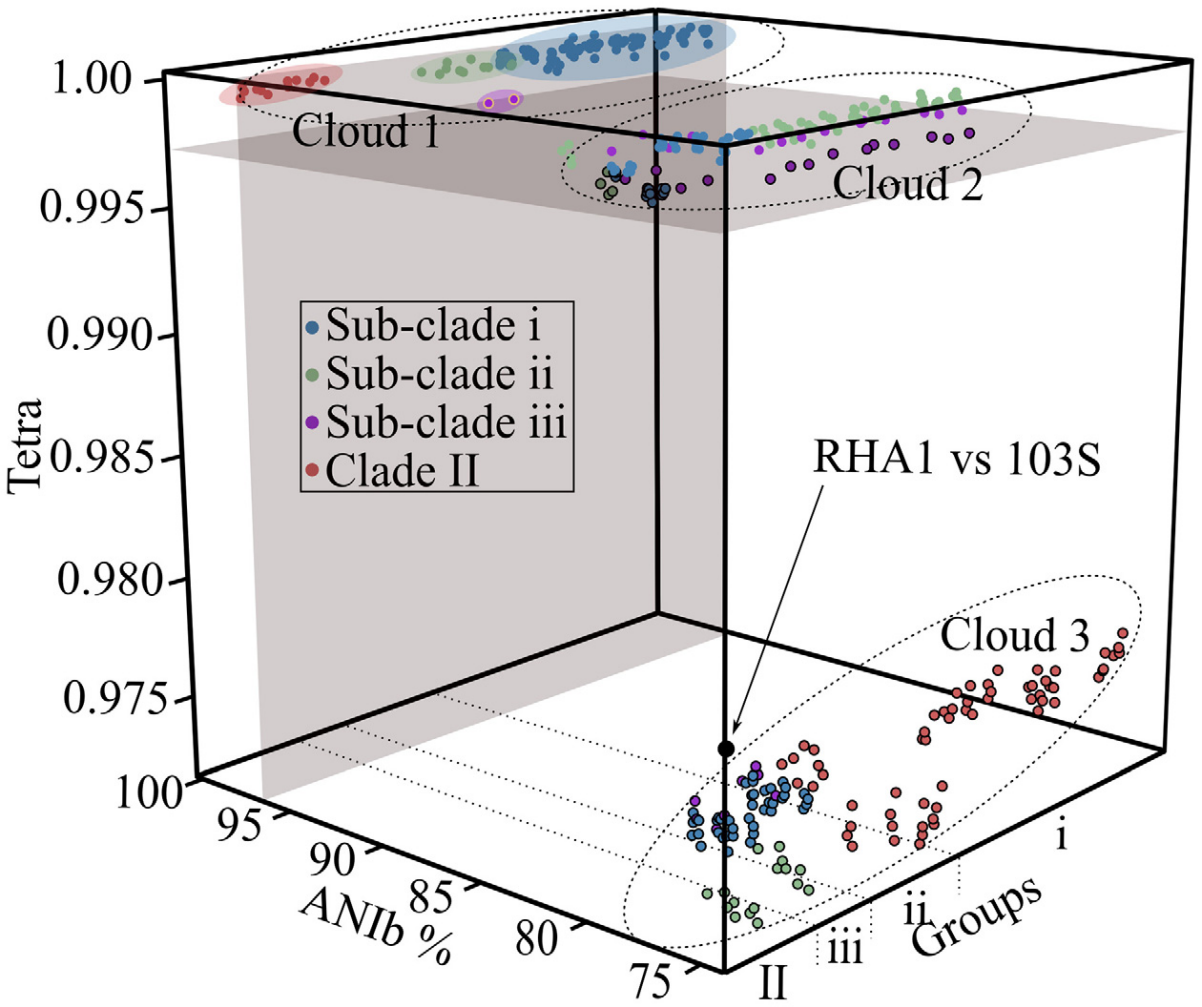
**Cloud plot of ANIb vs. TETRA for all possible pairwise comparisons.** Average nucleotide identity (ANIb; x-axis) and tetranucleotide (TETRA; y-axis) usage patterns were calculated and plotted as a factor of isolate grouping (z-axis). All pairwise comparisons, including reciprocal comparisons are presented, with colors assigned based on the clade membership of the isolate being compared to. Gray colored areas demark 96% ANI and 0.997 TETRA thresholds. Clouds are demarcated by dotted lines. Circles with black borders are below the TETRA threshold. Circles with yellow borders (comparisons between isolates of sub-clade iii; purple) exceed the TETRA threshold but not the ANIb threshold. The black circle represents a comparison between *R. jostii* RHA1 and *R. equi* 103S.

To support the existence of distinct groups of plant pathogenic *Rhodococcus*, we used alternative methods to cluster the bacteria based on similarities in their genome sequence features. We determined the average nucleotide identity values (ANIb; calculated with the BLAST algorithm) and tetranucleotide usage patterns (TETRA) for all pairwise comparisons (Teeling et al., 2004; Konstantinidis and Tiedje, 2005; Richter and Rosselló-Móra, 2009). A plot of ANI vs. TETRA formed three distinct clouds (Figure 2). The plots of the comparisons of isolates within sub-clades i and ii as well as Clade II coalesced into cloud 1. These comparisons were wholly within the calibrated and strictest thresholds of 96% ANI and 0.997 TETRA values that are recommended for circumscribing prokaryotic taxa (Richter and Rosselló-Móra, 2009). Results were identical when we relaxed ANI thresholds to 94% (data not shown). The reciprocal comparisons between isolates 05-339-1 and A76 of sub-clade iii associated with Cloud 1 but fell below ANI thresholds, regardless of which strictness level was used. The failure to exceed threshold is consistent with the greater divergence between these isolates, as observed in the NJ tree. Cloud 2 represented all possible comparisons between isolates in different sub-clades of Clade I. This cloud spanned the TETRA threshold value but was well below the ANI threshold values (Richter and Rosselló-Móra, 2009). Cloud 3 contains the most dissimilar comparisons between isolates of Clades I and II. The values within this cloud are similar to those derived from a comparison between *R. equi* 103S and an environmental isolate of *Rhodococcus* (McLeod et al., 2006; Letek et al., 2010). Thus, not only was the structure of the *Rhodococcus* samples supported by analysis with ANI and TETRA, but the consistency in results reconfirmed the use of ANI for inferring genetic relatedness, as was previously demonstrated by others (Goris et al., 2007; Richter and Rosselló-Móra, 2009).

**Figure 3:**
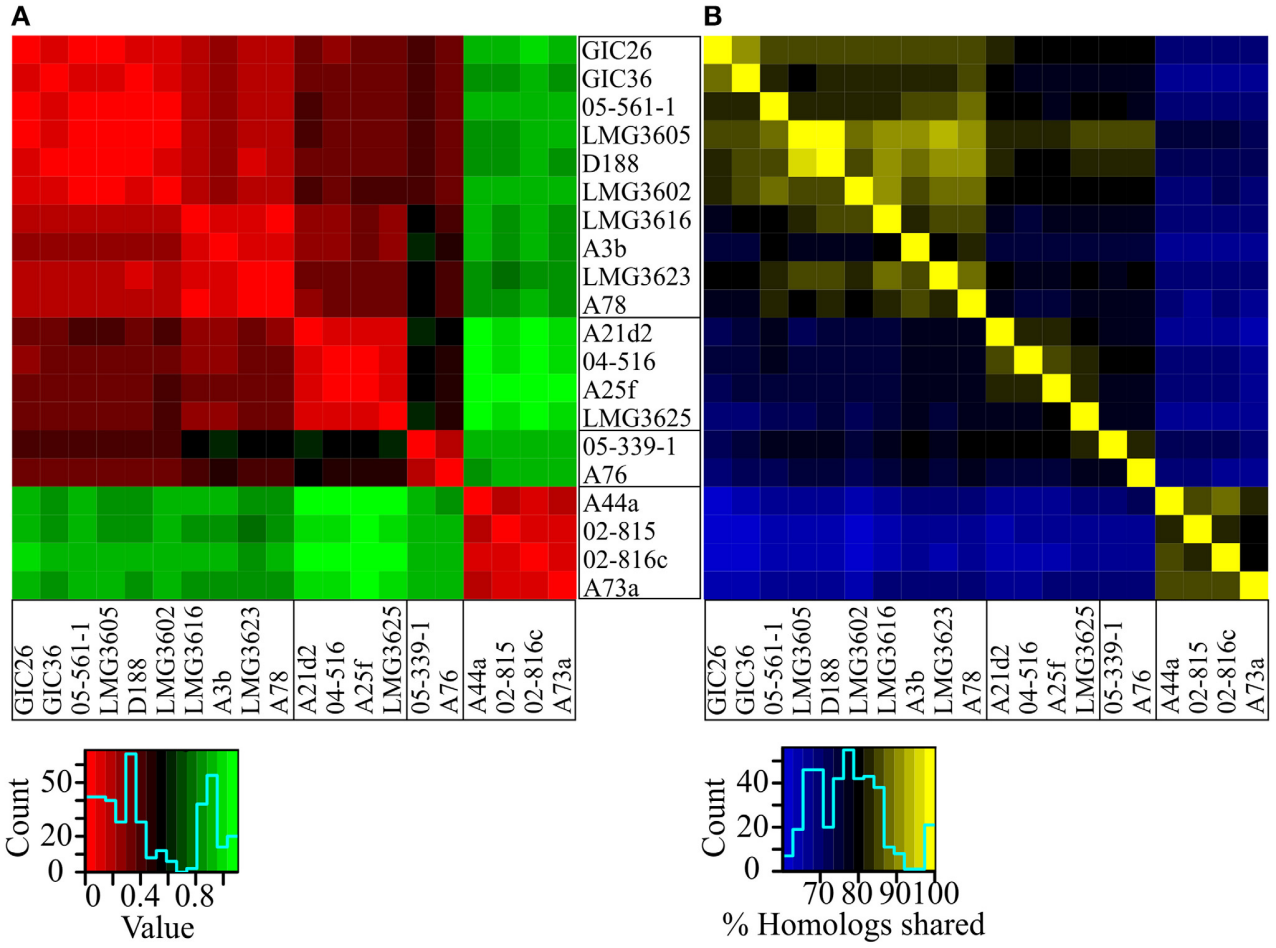
**Heatmaps of codon usage preference and homology. (A)** Codon usage preference similarities were calculated for all possible pairwise comparisons. Lower values indicate fewer differences. Isolates were ordered according to their phylogenetic relationships. **(B)** Reciprocal BLASTP analysis was used to determine percent homology for all possible pairwise comparisons. Larger values indicate greater similarities. Isolates were ordered according to their phylogenetic relationships.

The genetic code is nearly universal but synonymous codons are not used with equal frequencies across species because of a complex balance of multiple forces (Plotkin and Kudla, 2011). Because this codon bias can be used to distinguish between dissimilar groups of organisms, we compared all possible pairwise combinations between the 20 isolates and displayed the similarity values as a heat map (Sharp and Li, 1987). Codon usage preferences clearly differentiated the isolates of Clade I from those of Clade II (Figure 3A). Codon usage preferences also revealed a pattern consistent with the substructure observed in the NJ tree and ANI vs. TETRA analysis (Figures 1, 2). The three sub-clades were easily discernable, though the relationships within sub-clade i differed slightly from those inferred from the NJ tree.

The core genome hypothesis suggests that coherent clusters of bacteria have a core set of functions, and each member augments the core with a variable accessory genome that contributes functions for niche adaptation (Lan and Reeves, 1996). The 20 sequenced isolates share a core of 3063 genes. However, sample size indubitably affects estimation of core genome identities and sizes. Given the small sample size of our collection and the imbalance in numbers of isolates between clades, we elected to cluster based on the percent of shared homologs rather than on core genomes (Figure 3B). The members of Clades I and II separated into distinct clusters, which could be taken as evidence for limited gene flow between clades. Relative to results from other approaches, the relationship of the isolates within Clade I were noticeably different, as sub-clade ii and sub-clade iii were not clearly demarcated. Regardless of these subtle differences, the results were entirely consistent in separating the 20 isolates into two groups of phytopathogenic *Rhodococcus*, with one also having evidence for substructure.

### A molecular phylogeny based on whole genome sequences provides a framework for resolving the *Rhodococcus* genus

**Figure 4:**
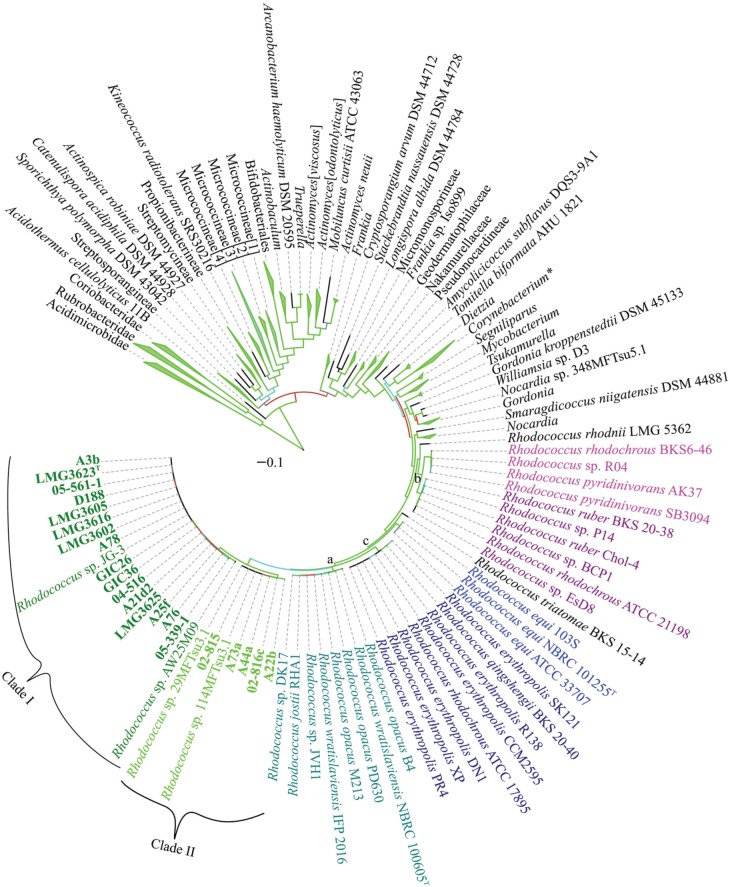
**Multi-locus sequence analysis maximum likelihood tree of the Actinobacterium phylum.** Translated sequences for *ftsY*, *infB*, *rpoB*, *rsmA*, *secY*, *tsaD*, and *ychF* from 1316 members were identified using TBLASTN, aligned, and used to generate a multi-locus maximum likelihood tree. A total of 961 sequences were used as input for tree generation. The 21 *Rhodococcus* isolates sequenced by our group are shown in bold and the two clades that include phytopathogenic *Rhodococcus* spp. are indicated. The *R. erythropolis*, *R. rhodochrous*, and *R. equi* clades, previously identified based on a 16S rDNA phylogeny of *Rhodococcus* are labeled with a, b, and c, respectively. Type strains are indicated with a superscript “T.” Branches outside of the *Rhodococcus* genus were collapsed at the genus, family, suborder, order, and subclass level, as appropriate, with the corners of the triangle indicating the shortest and longest total branch lengths for the members of the collapsed clade. A total of 500 rapid bootstraps were performed on this dataset, and branches are colored on a gradient to indicate bootstrap percentage (Green-Cyan-Red, with cutoffs of 100-75-50 and below, respectively). Scale bar, mean number of amino acid substitutions per site.

To develop a framework for resolving the *Rhodococcus* phylogeny, we constructed a multi-locus sequence analysis (MLSA) maximum likelihood (ML) tree based on 961 concatenated sequences representing 1316 members of the Actinobacteria phylum (Figure 4). We used seven marker genes that were previously identified as conserved and informative for the subclass Actinobacteridae (Adékambi et al., 2011). *Rhodococcus* and *Nocardia* formed sister groups and the members of the *Rhodococcus* genus formed a well-supported phylogenetically coherent cluster (bootstrap percentage of 98%). We were able to identify two relatively defined groups and one small, less defined, group within the *Rhodococcus* genus (Figure 4; marked as a, b, and c). The two larger groups were consistent with the two clades previously described in a phylogeny based on 16S rDNA sequences (Jones and Goodfellow, 2012). In contrast, in the MLSA ML tree, the smaller *R. equi* clade is within the larger *Rhodococcus* clade (bootstrap percentage of 93%), unlike previous studies, which associated the *R. equi* clade with *Nocardia*.

The 20 isolates of interest in this study formed a distinct subgroup (bootstrap percentage of 100%) within the MLSA ML tree and also included five other isolates (Figure 4). Phytopathogenic isolate A22b, which was identified from a diseased plant and sequenced independently from the 20 isolates, clustered in Clade II. Two of the isolates that clustered with the 20 of interest in this study, were identified independent of plants and in extreme environments. *Rhodococcus* sp. JG-3 was isolated from permafrost (GenBank BioProject PRJNA195882) and *Rhodococcus* sp. AW25M09 was isolated from the stomach of an Atlantic Hagfish (Hjerde et al., 2013). Two others, *Rhodococcus* spp. 29MFTsu3.1 and 114MTsu3.1 were found associated with the rhizosphere or endosphere of *Arabidopsis thaliana* (GenBank BioProject PRJNA201196).

The topology of the tree outside of the *Rhodococcus* genus was similar to previously reported trees and revealed inconsistencies in currently defined taxonomic groups, as previously observed (Adékambi et al., 2011; Gao and Gupta, 2012; Jones and Goodfellow, 2012; Verma et al., 2013). The Micrococcineae formed a polyphyletic group, with three to four distinct clades, depending on the tree and tree generation method (bootstrap percentages of 88–100%). We also had difficulties in accurately placing the Frankineae into a discrete group, as they were found throughout the phylogeny. The *Actinomyces neuii* species formed a separate, but somewhat poorly supported (bootstrap percentage of 63%) clade with *Mobiluncus curtisii*, as was the case in a phylogeny based on 16S rDNA sequences (Jones and Goodfellow, 2012). We observed a cryptic relationship with Actinopolysporineae included within the Pseudonocardineae clade. However, some of the branches within the Pseudonocardineae were poorly supported (bootstrap percentages <50%), indicating poor resolution with the clade as a whole. One key addition of the MLSA ML tree was that the branching of *Actinomyces* was well supported and is consistent with there being two large clades (bootstrap percentages of 90 and 100%).

### ANI provides a framework for resolving the *Rhodococcus* genus

**Figure 5:**
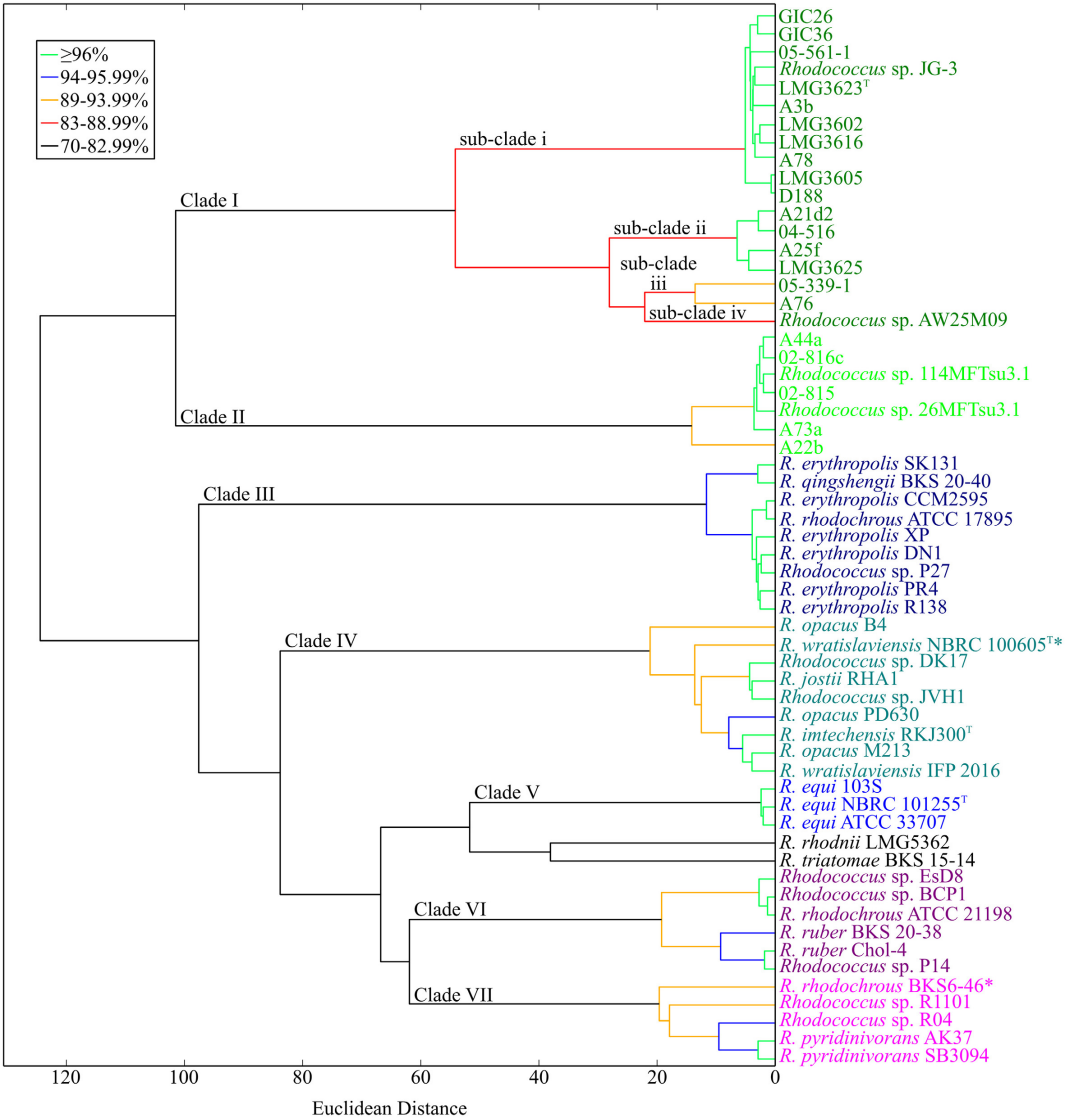
**Average nucleotide identity dendogram for 59 isolates of *Rhodococcus*.** Complete genome sequences for 59 isolates of *Rhodococcus* were used to generate an ANI matrix. The matrix was used to calculate an ANI divergence dendrogram. Groups are color coded according to groups represented in the MLSA ML tree. Branches are colored using cutoffs for pairwise comparisons of all taxa after the nodal point. The 21 *Rhodococcus* isolates sequenced by our group are shown in bold; type strains are designated with a superscript “T.” Clades and sub-clades of the phytopathogenic *Rhodococcus* isolates are labeled at the corresponding node. ^*^Indicates conflict between placement within the dendogram and calculated ANI values (see corresponding text for details).

Because of the relatively few informative sites, bootstrap values at the tips of the MLSA ML tree were often low and insufficient for resolving the *Rhodococcus* genus; compare for example, the two clades of *Rhodococcus* with plant pathogenic members (Figures 1, 4). Therefore, to develop a molecular framework for the *Rhodococcus* genus, we used ANIb as a tool for inferring similarity. The values were calculated for 3422 pairwise comparisons of the 59 *Rhodococcus* isolates, compiled into a distance matrix, and used to generate a divergence dendogram (Figure 5; Chan et al., 2012). Seven distinct clusters formed with inter-group comparisons that exceeded ANI values of 70–75%, a range that is typically found between members of the same genus.

The two clades of phytopathogenic *Rhodococcus* spp. represent seven different species, when using ANI and a 94% threshold. As previously observed, Clade I has complex substructure and represents four different species (Figures 1–3, 5). Like conclusions described above, subclade i represents the originally named *R. fascians* species, as it includes the type strain LMG3623, along with the most sequenced isolates. Isolates in subclade iii were not as closely grouped, which was also consistent with results from the NJ tree and analysis of codon usage. In fact, based on the single criterion of ANI, 05-339-1, and A76 should be considered as separate species. Subclade iv is represented by a single isolate, *Rhodococcus* sp. AW25M09. In Clade II, isolate A22b is just below the 94% ANI cutoff and may represent a separate subspecies from those that formed this second major clade in previously described analyses (Figures 1–3, 5).

The other isolates of *Rhodococcus*, which are not known as plant-associated, formed five additional clades. Within these clades, and including single isolates (singletons), there were 13 smaller groups defined by ANI values of 94% ANI or greater. These relationships can be used to infer species groups.

Clade III consists mostly of isolates named as *Rhodococcus erythropolis*. The group also included *Rhodococcus qinshengii* BKS 20-40, *Rhodococcus rhodochrous* ATCC 17895, and *Rhodococcus* sp. P27, which based on their association to the clade, could be considered members of the *R. erythropolis* species.

Clade IV has two subgroups and two singletons. The first subgroup is represented by *Rhodococcus jostii* RHA1 and also included *Rhodococcus* spp. DK17 and JVH1. The second subgroup varied in terms of named species and consisted of two isolates of *Rhodococcus opacus*, the type strain of *Rhodococcus imtechensis* and *Rhodococcus wratislaviensis* IFP 2016. Because the latter strain did not cluster with the type strain of *R. wratislaviensis*, NBRC 100605, we suggest that IFP 2016 is not a member of the *R. wratislaviensis* species and it belongs to a different species of *Rhodococcus*. The other singleton isolate was *R. opacus* B4. A whole genome sequence of the *R. opacus* type strain is unavailable. Thus, we cannot suggest whether the subgroup or the singleton should be designated as the *R. opacus* species. Furthermore, 16S rDNA sequences of the three named *R. opacus* isolates are too similar for resolving this issue. Interestingly, *R. opacus* PD630 is most similar to *R. wratislaviensis* NBRC 100605 in respect to ANI values, but the two did not associate with one another in the divergence dendogram.

Clade V contains a tightly clustered group with members of the *Rhodococcus equi* species. Its placement within *Rhodococcus* was consistent with the MLSA ML tree.

Clade VI consists of two smaller subgroups. The first subgroup has *R. rhodochrous* ATCC 21198 and two undesignated isolates (EsD8 and BCP1). The low ANI of 72% placed *R. rhodochrous* ATCC 21198 in a clade separate from isolate *R. rhodochrous* BKS6-46. To further investigate this discrepancy, we used the 16S rDNA sequence from *R. rhodochrous* ATCC 21198 as a query in a BLASTN+ search. The sequence identified corresponding sequences from *Rhodococcus aetherivorans* (100% identity, 100% subject coverage), including the type strain DSM 44752. When we used the 16S rDNA sequence from the type strain of *R. rhodochrous* as a query, it showed greater similarity to the corresponding sequence of *R. rhodochrous* BKS6-46 rather than ATCC 21198. In all, these data suggest the identity of ATCC 211983 should be revisited. The second subgroup of Clade VI consists of two *Rhodococcus ruber* isolates (BKS 20-38 and Chol-4) and another undesignated isolate (P14). Thus, we suggest that P14 is a member of the *R. ruber* species.

Clade VII has one cluster of defined species and two singletons. Two isolates of *Rhodococcus pyridinivorans* (AK37 and SB3094) and *Rhodococcus* sp. R04 clustered, which we suggest represents the *R. pyridinivorans* species. *R. rhodochrous* BKS6-46 is a singleton in this clade but its precise placement within this clade of the dendrogram is somewhat misleading. The ANI values for the pairwise comparisons of *R. rhodochrous* BKS6-46 to the two named *R. pyridinivorans*, but not *Rhodococcus* sp. R04, exceeded the 94% threshold used to define a species relationship. The values derived from comparison with *Rhodococcus* sp. R04 likely caused *R. rhodochrous* BKS6-46 to form its own branch. The last singleton is *Rhodococcus* sp. R1101, which had ANI values around 90% relative to the other isolates of Clade VII.

There were two outliers in the dendogram. *Rhodococcus rhodnii* and *Rhodococcus triatomae* were identified as singletons and placed in a clade closest to Clade V (*R. equi*). These isolates had low ANI values (between 71 and 75%) in comparison to all of the sequenced *Rhodococcus* isolates.

## Discussion

Leafy gall disease is a substantial economic problem for the horticultural industry. The pathogen has an extensive host range that includes most plants important to the industry (Putnam and Miller, 2007). Current management strategies rely on visual inspection and the only method of control is the destruction of infected plant material. While visual inspection of plants for disease is superficially trivial, the absence of fundamental information on its epidemiology and the lack of robust, on-site diagnostics contribute to make disease management challenging. Whole genome sequencing is a cost-effective and facile approach for studying bacterial species and has important practical implications for diagnosis of disease. In this study, we used whole genome sequences to analyze the genetic diversity of plant pathogenic *Rhodococcus* as a first step toward the development of better management strategies for this pathogen.

Twenty isolates, many of which were identified from diseased plants, were previously selected for whole genome sequencing (Creason et al., 2014). Based on a tree derived from 1727 homologous genes, we demonstrated that the 20 isolates separated into distinct clades and sub-clades (Figure 1). Similarities in genome features, including ANI, TETRA, codon usage preference, and degree of genome homology, were all consistent in clustering the isolates into distinct and coherent groups (Figures 2, 3). The similarity in results between the tree and ANI was encouraging and gave us confidence in using the latter for inferring evolutionary relationships of isolates within a larger dataset (Figure 5).

The phytopathogenic isolates formed a subgroup distinct from other members of the *Rhodococcus* genus, which could be taken as evidence for cohesion (Figures 4, 5). However, within this subgroup, phytopathogenicity is not a discriminative trait (Figures 1, 4, 5). Although, we speculate that members of this subgroup are potentiated toward phytopathogenicity. Virulence evolution in *Rhodococcus* has been modeled according to a mechanism of gene co-option whereby limited, but key horizontally acquired virulence genes, trigger the co-option of core genes for virulence (Letek et al., 2010; Creason et al., 2014). As few as four functions, most often conferred by a cluster of genes vectored by a conjugative virulence plasmid, are hypothesized to be sufficient for phytopathogenicity for members of these genetically diverse clades of *Rhodococcus*.

The non-pathogenic isolates recovered from a Greenland glacier ice core, GIC26, and GIC36, as well as *Rhodococcus* sp. JG-3 from permafrost and AW25M09 from the stomach of the Atlantic Hagfish, clustered with phytopathogenic isolates in Clade I (Miteva et al., 2004; Hjerde et al., 2013). *Rhodococcus* spp. 29MFTsu3.1 and 114MTsu3.1, both of which associate with plants, clustered in Clade II. Inspection of the genome sequences of isolates JG-3, AW25M09, 29MFTsu3.1, and 114MTsu3.1, failed to reveal any of the virulence genes known to be necessary for *Rhodococcus* to cause leafy gall disease. We did detect a linear plasmid-like sequence in the draft genome of *Rhodococcus* sp. AW25M09, but it lacks genes known to be necessary for virulence toward plants (Hjerde et al., 2013). It would be interesting to test whether these isolates, upon acquisition of genes that confer the four key virulence functions, gain the ability to infect and cause disease to plants.

Our genome sequencing effort of plant-associated isolates contributed to increasing the number of sequenced *Rhodococcus* isolates by 50%. Not since a decade ago, have genomic features been used to address the *Rhodococcus* genus (Gürtler et al., 2004). We therefore used these genome sequences, an additional sequence we generated for phytopathogenic isolate A22b, along with most currently available rhodococcal genome sequences, to construct a molecular phylogeny to help resolve the genus and shed light on the phylum Actinobacteria as a whole. MLSA provided a framework for defining genus level relationships that can be further explored (Figure 4). In this study, the *R. equi* species was placed within the *Rhodococcus* genus, consistent with previously reported results derived from whole-genome based approaches, and in contrast to phylogenetic analysis based on 16S rDNA sequences (Letek et al., 2010; Jones and Goodfellow, 2012). Use of MLSA to infer phylogeny revealed inconsistencies in the placement of Frankineae, in contrast to a 16S rDNA-based phylogeny, which formed a Frankineae cluster near the root of the phylum (Jones and Goodfellow, 2012). However, MLSA was not sufficient for resolving some of the branches within the Pseudonocardineae (bootstrap percentages <50%). More genome sequences, or informative marker sequences for members of Actinopolysporineae might help discern the finer details within the suborder. Other minor discrepancies in single isolate naming were also noted (Supplemental Data 1).

With ANI, we were able to place the 59 *Rhodococcus* isolates with sequenced genomes into seven large groups. Results were consistent with the MLSA ML tree but ANI provided greater resolution of their relationships (Figure 5). We were also able to infer at least 20 different species from these 59 isolates. Some conflicts between clustering and species naming were noted and suggest a need to revisit their taxonomical groupings. However, we recognize that the use of ANI to infer taxonomical groupings merely provides a framework and is not sufficient, by itself, for defining bacterial species. At present, in order to validly designate a species, it needs to be further characterized for at least one discriminative trait. Moreover, the designation of a species would also require inclusion of their corresponding type strain.

The methods that were used were congruent in supporting the existence of multiple species of *Rhodococcus*. However, each of the methods has limitations. The tree developed based on whole genome sequences was computationally and time intensive and precluded us from using methods with stronger statistical frameworks for phylogenetic reconstruction and also became too time-intensive for larger datasets. MLSA is limited by the need for a sufficient set of generalizable and informative marker genes. Despite the reduced number of sequences, the MLSA approach was still computationally and time intensive with larger datasets. ANIb can be limiting in projecting relationships in an evolutionary context (Figure 2). Though the dendrogram constructed based on Euclidean distances was a convenient way for visualizing dissimilarities, there were nevertheless some discrepancies between placement of isolates and their measured ANI values (Figure 5). For example, *R. opacus* PD630 and *R. wratislaviensis* NBRC 100605 have an ANI value that warranted consideration as a species, but their dissimilarities to other isolates prevented the two from clustering in the dendogram (Figure 5). Lastly, similarities based on codon usage preferences and genome inventories were inadequate for resolving relationships between isolates with highly similar genome signatures (Figure 3). Nevertheless, when multiple methods were coupled, we were able to make strong inferences regarding taxonomical relationships.

In summary, we used whole genome sequences to resolve the phytopathogenic members of *Rhodococcus* into multiple sister species and developed a dataset that contributes to reconstructing the phylogeny of the *Rhodococcus* genus.

## Author contributions

These authors contributed equally. Allison L. Creason, Edward W. Davis II, Melodie L. Putnam, Olivier M. Vandeputte and Jeff H. Chang contributed to the conception and design of the work, analysis of data, and wrote and approved the manuscript. Allison L. Creason, Edward W. Davis II, Melodie L. Putnam, and Olivier M. Vandeputte also contributed to the acquisition of isolates and/or genome sequences. The authors are accountable for all aspects of the work.

### Conflict of interest statement

The authors declare that the research was conducted in the absence of any commercial or financial relationships that could be construed as a potential conflict of interest.

## Supplementary material

Figure S1 **Genome alignments of four members of phytopathogenic *Rhodococcus*.** Sequences of the chromosomes of isolates D188, A21d2, 05-339-1, and A44a were aligned using progressiveMauve. Each colored square represents a block of sequences that is collinear to a corresponding block of sequences in another genome sequence; linear collinear blocks (LCBs). The extent of homology within LCBs is represented by the height of the colored plot in each block.

Supplemental Data 1 **Compressed folder with MLSA tree in Newick tree format file and its associated data.** The phylogenetic tree of the entire Actinobacteria phylum is available as a Newick file. Also included are three comma separate files. (1) SD1_identical_seqs.csv: lists the names of the duplicate sequences that were removed prior to generating the final tree. The names of the species used in generating the tree are listed in the first column. The remaining columns contain the names of the species that were filtered out based on having an identical sequence. (2) SD1_names_identifiers.csv: associates genome sequence identifiers (either GI number for complete genomes, or 4 letter code for wgs sequences) to corresponding isolates in the tree. (3) SD1_taxonomy.csv: lists the taxonomy values for each of the taxa.

Supplemental Data 2 **Compressed folder with Python script and input data for ANI dendrogram generation.** A Python script file is included that can generate an ANI dendrogram from the two input files, one that includes the ANI divergence matrix (SD2_ani_data.txt), and one that includes the names of the genomes included in the analysis (SD2_ani_names.txt).
