# Supplementary figures and images for: Use of whole genome sequences to develop a molecular phylogenetic framework for Rhodococcus fascians and the Rhodococcus genus
Source: Front Plant Sci. 2014 Aug 19;5:406. doi: 10.3389/fpls.2014.00406 (PMC4154481; doi:10.3389/fpls.2014.00406)

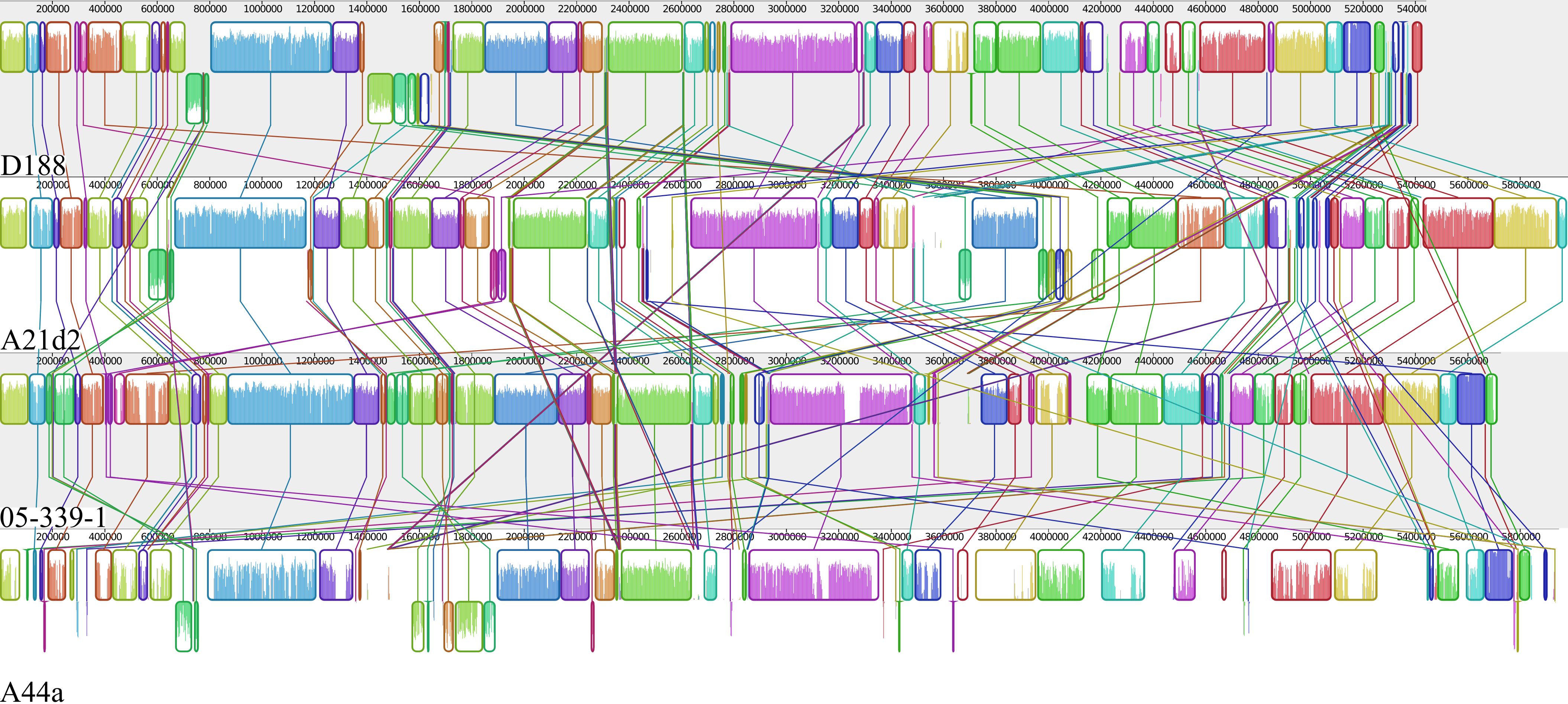

Supplement: Figure S1 — Genome alignments of four members of phytopathogenic Rhodococcus. Sequences of the chromosomes of isolates D188, A21d2, 05-339-1, and A44a were aligned using progressiveMauve. Each colored square represents a block of sequences that is collinear to a corresponding block of sequences in another genome sequence; linear collinear blocks (LCBs). The extent of homology within LCBs is represented by the height of the colored plot in each block. [file Image1.JPEG]
